# Interplay of missed opportunity for vaccination and poor response to the vaccine led to measles outbreak in a slum area of Eastern Mumbai, India

**DOI:** 10.1017/S0950268824000426

**Published:** 2024-03-18

**Authors:** Reetika Malik Yadav, Mangala Gomare, Arun Gaikwad, Upalimitra Waghmare, Utkarsh Betodkar, Meeta Dhaval Vashi, Vineet Kumar Kamal, Jeromie Wesley Vivian Thangaraj, Sampada Bangar, Tarun Bhatnagar, Manoj Murhekar

**Affiliations:** 1 ICMR School of Public Health, ICMR-National Institute of Epidemiology, Chennai, India; 2 Department of Pediatric Immunology, ICMR-National Institute of Immunohaematology, Mumbai, India; 3 Public Health Department, Municipal Corporation of Greater Mumbai, Mumbai, India; 4 World Health Organization, Mumbai, India; 5 Division of Epidemiology and Statistics, ICMR-National AIDS Research Institute, Pune, India

**Keywords:** failure to vaccinate, measles, outbreak, surveillance, vaccine failure

## Abstract

In the third week of September 2022, an outbreak of measles was reported from a slum in Eastern Mumbai, India. We sought to investigate whether failure to vaccinate or vaccine failure was the cause. We constructed an epidemic curve, drew a spot map, and calculated the attack rate and case-fatality ratio. We calculated vaccine effectiveness (VE) for one and two doses of measles vaccine in an unmatched case–control study and did stratified analysis by sex, availability of vaccination card, and migrant status. We identified 358 cases and four deaths with a 11.3% attack rate and 1.1% case fatality, both being highest among 0–24-month-old boys. The epidemic curve suggested a propagated mode of spread. The VE for two doses was 64% (95% confidence interval (CI): 23–73%) among under-5-year-old children and 70% (95% CI: 28–88%) among 5–15-year-old children. Failure to vaccinate, consequent to the COVID-19 pandemic, and vaccine hesitancy might have led to the accumulation of susceptible children in the community. Additionally, the occurrence of case-patients among vaccinated suggests reduced VE, which needs further investigation into humoral and cell-mediated immunity as well as contributory factors including nutritional status. Outbreak response immunization to complete immunization of missed and dropout children was carried out to control the outbreak.

## Introduction

India has committed to measles elimination by 2023 [1]. Efforts are being made towards the elimination goal by achieving and sustaining vaccination coverage of 95% with two doses of a measles-containing vaccine (MCV) at the national and sub-national levels. As per the immunization schedule of India, the first dose of MCV is administered between 9 and 12 months of age and the second between 16 and 24 months [2]. Supplementary immunization with MCV irrespective of vaccination status was also conducted in 2018–2019. Case-based surveillance and an established network of 27 laboratories across the country aid in the country’s efforts towards elimination. India achieved the global standard non-measles non-rubella discard rate of 2 per 100,000 population during the last six months of 2021 [3].

In 2022, India had recorded 12,773 measles cases till November [4]. India topped the Centers for Disease Control and Prevention (CDC) global list of countries with measles outbreaks from November 2022 to April 2023 [5]. Measles cases were reported from the states of Maharashtra [6, 7], Gujarat [8], Kerala [9], Uttar Pradesh [10], and Madhya Pradesh [11] from the latter part of 2022 with Maharashtra being the worst affected [8].

Between 17 and 23 September 2022, six suspected measles cases were reported from a slum area of Eastern Mumbai, India, predominantly belonging to a religious minority. Measles incidence in Eastern Mumbai in 2022 showed an upward trend with 0.83/million population compared to 0 in the previous three years. An outbreak was declared on 20 September 2022 after the detection of measles IgM antibodies by enzyme-linked immunosorbent assay (ELISA) in five of the six reported case-patients. We investigated the outbreak to describe its epidemiology, ascertain measles vaccination status, and provide recommendations for its control.

## Methods

### Case definitions

We defined a suspect case [12] as the occurrence of any maculopapular rash illness with fever in a person of any age in the slum area of Eastern Mumbai between 17 June 2022 and 20 February 2023 for a house-to-house case search. We further classified these cases as probable and laboratory-confirmed (immunoglobulin M (IgM) ELISA-positive) cases. We defined a measles death as death within 30 days of onset of rash without any obvious causes like trauma, accident, or snake bite in a person of any age within the last 90 days [12].

### Descriptive epidemiology

Mumbai City Municipal Corporation initiated an active house-to-house search for cases immediately after the outbreak confirmation in the Eastern Mumbai slum, which continued till February 2023 when the outbreak was declared closed. During the active case search, data were collected on the demography, symptoms, vaccination status for MCV, laboratory testing, and results for suspect cases on a paper-based form and compiled in Google Sheets. We constructed an epidemic curve by plotting the distribution of cases by the date of onset of fever. We drew a spot map of the cases among the households in the slum area. We obtained the age and sex distribution of the under-5 child population and the 5–15-year population from the municipal corporation for the calculation of attack rates and case fatalities by age and sex.

### Analytical epidemiology

We hypothesized that failure to vaccinate or vaccination failure of the MCV could be associated with the outbreak. To test these hypotheses, we conducted an unmatched case–control study, separately for the under-5 and 5–15-year age groups among the households in the outbreak area. We defined a case as an individual having maculopapular rash illness with fever in the specified age groups in the last 90 days. We defined a control as an individual in the specified age group who did not satisfy the criteria for a case. Cases and controls were enrolled from different households.

Using the OpenEpi software, we calculated a sample size of 133 cases and 133 controls each in the under-5 and 5–15-year age groups assuming an odds ratio (OR) of 0.5 associated with vaccination, 57% of cases vaccinated with one dose of MCV (unpublished data from Mumbai Municipal Corporation), a 95% confidence interval (CI), and a 1:1 case–control ratio.

We collected information from suspected measles cases and controls about the receipt of routine and supplemental measles vaccination along with reasons for not vaccinating, if unvaccinated, and duration of residence in Mumbai using a structured form by interviewing the caregiver of the children through face-to-face interviews and reviewing the vaccination cards. Wherever a vaccination card was not available, a recall of vaccination administration was asked from the caregiver.

We calculated the OR for one and two doses of MCV and vaccine effectiveness (VE) as VE = 1 – OR separately for the under-5 and 5–15-year age groups. We also performed a stratified analysis by sex, availability of vaccination cards, and migrant status (defined as less than ten years of residence in Mumbai). We used Epi Info version 7.2 for the statistical analysis.

## Results

### Description of the outbreak

We identified 358 cases (including six laboratory-confirmed) and four deaths among the under-15 population of 3,156 residing in approximately 3,100 houses in the slum, with an overall attack rate of 11.3% and 1.1% case fatality. Attack rates were highest in the 0–24-month age group (38.8%) and boys (13%) (Table 1). The median age of case-patients was 30 months (interquartile range (IQR) 16–48 months). Cough, coryza, conjunctivitis, and pneumonia were seen in 94%, 94.3%, 51.6%, and 0.8% of case-patients, respectively, at first presentation.

**Table 1.** Distribution of case-patients by age and sex, Eastern Mumbai slum, India, September 2022–February 2023

Person characteristics		Number of cases	Population (*n*)	Attack rate (%)	Number of deaths	Case fatality (%)
Age (in months)	0–12	76	234	32.5	0	0
	13–24	85	219	38.8	2	2.4
	25–60	159	648	24.5	2	1.2
	61–120	32	986	3.2	0	0
	120–180	6	1,069	0.6	0	0
Sex	Male	205	1,582	13	4	2
	Female	153	1,574	9.7	0	0
Total		358	3,156	11.3	4	1.1

**Figure 1.**
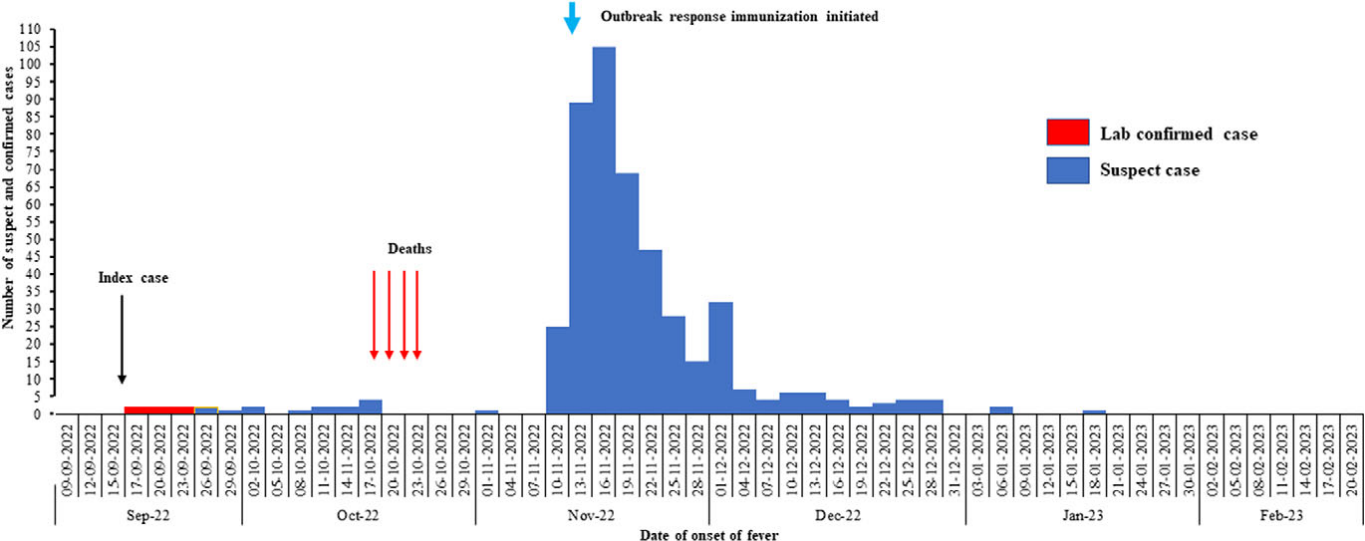
Distribution of cases by date of onset of fever in Eastern Mumbai slum, India, September 2022–February 2023.

**Figure 2.**
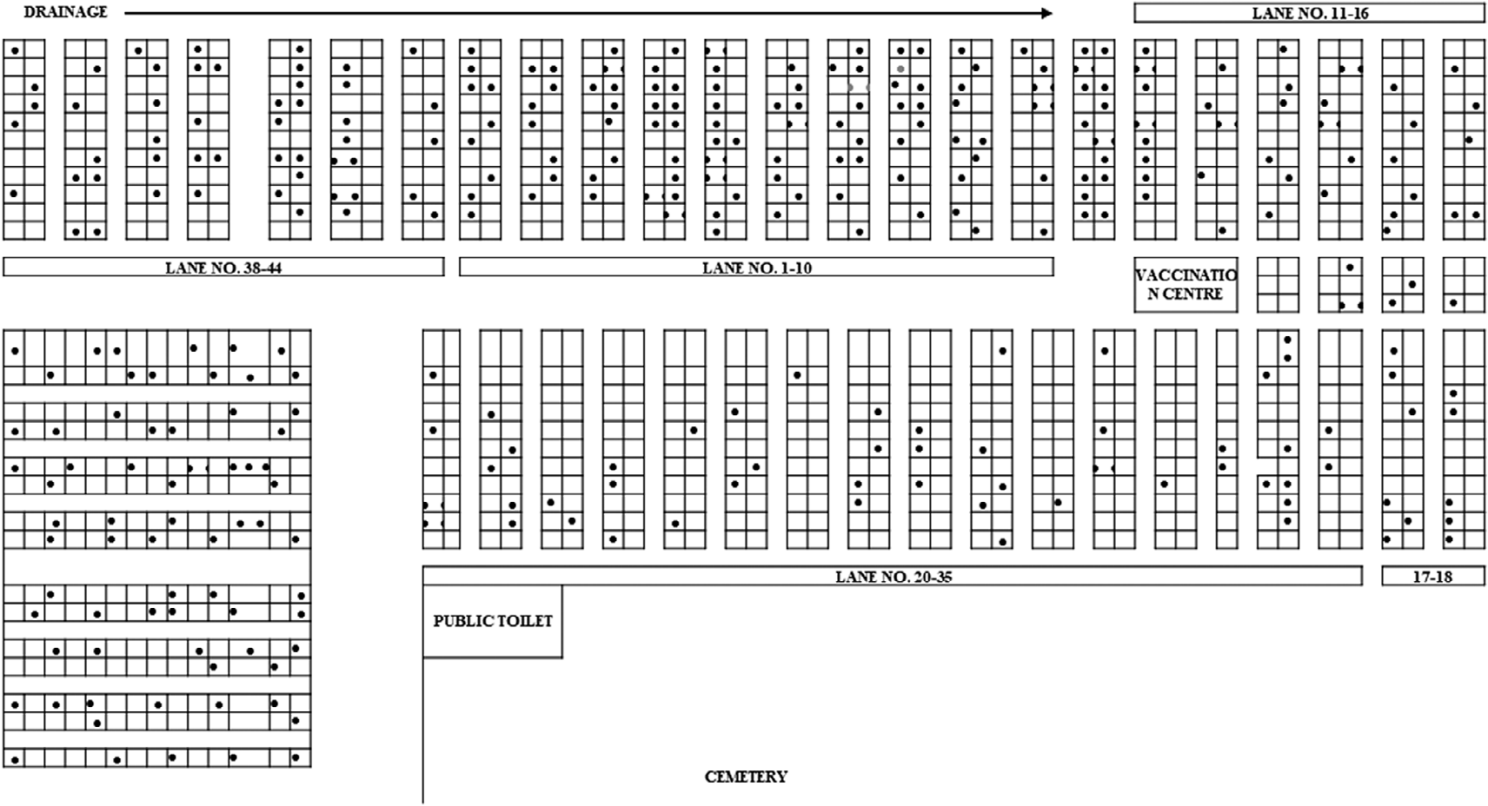
Distribution of cases by place in Eastern Mumbai slum, September 2022–February 2023.

The index case had febrile rash illness in the third week of September 2022. Majority of the case-patients reported onset of fever in the second week of November 2022 (*n* = 132, 36.9%) and peaked by the end of the second week. Thereafter, the number of cases declined. All four measles deaths occurred before the first peak. Outbreak response immunization was initiated in the third week of November and coincided with the peak. A second smaller peak was seen in the first week of December 2022. The two peaks were one incubation period apart, suggesting propagated mode of spread through person-to-person transmission. The last case was reported during the second week of January 2023 (Figure 1). Cases were distributed across the slum area (Figure 2).

Among controls, 117 (71%) and 82 (63%) among the under-5 age group and 85 (88%) and 64 (82%) among the above-5 age group had received one and two doses of MCV, respectively. Overall, 46 (16.3%), 149 (51.9%), and 91 (31.7%) case-patients reported receiving zero, one, and two doses of MCV, respectively. Despite supplementary immunization activities in 2018–2019, none of the case-patients reported receiving three doses of MCV. Only about 50% of case-patients in the 9–12-month age group had received the first dose of MCV. 42.5% of male case-patients and 19% of female case-patients in the 13–24-month age group had received two doses of MCV. The percentage of case-patients vaccinated with either one or two doses of MCV was similar across both sexes except in the 13–24-month and above 120-month age groups where vaccination among female was lesser than among male case-patients (Table 2).

**Table 2.** MCV status of case-patients eligible for one and two doses by age and sex, Eastern Mumbai slum, India, September 2022–February 2023

Age (in months)	Number of case-patients (*N*)		Case-patients received zero dose of MCV *n* (%)		Case-patients received one dose of MCV *n* (%)		Case-patients received two doses of MCV *n* (%)	
	Male	Female	Male	Female	Male	Female	Male	Female
9–12^a^	37	35	18 (48.6)	16 (45.7)	19 (51.4)	19 (54.3)	NA	NA
13–24^b^	40	21	1 (2.5)	7 (33.3)	22 (55)	10 (47.6)	17 (42.5)	4 (19)
25–60	70	53	0 (0.0)	0 (0.0)	37 (52.8)	28 (52.8)	33 (47.1)	25 (47.2)
61–120	14	8	4 (28.6)	0 (0.0)	5 (35.7)	5 (62.5)	5 (35.7)	3 (37.5)
>120	6	3	0 (0.0)	1 (33.3)	3 (50)	1 (33.3)	3 (50)	1 (33.3)
Total	167	120	23 (13.8)	24 (20)	86 (51.5)	63 (52.5)	58 (34.7)	33 (38.8)

a Case-patients eligible for one dose of MCV only.

b Case-patients aged 16 months and above eligible for two doses of MCV.

The reasons reported by the caregivers for incomplete vaccination of children (*n* = 70) were the child being sick (59%), family-related problems (14%), lack of knowledge about immunization sessions (10%), adverse events following immunization (10%), lack of faith in vaccination (4%), and non-availability of staff for vaccination (3%).

### Vaccine effectiveness

VE was 40% (95% CI: 5–62%, *p*-value 0.43) and 64% (95% CI: 23–73%, *p*-value <0.01) for one and two doses of MCV, respectively, in the under-5 age group. The VE in the 5–15-year age group was 64% (95% CI: 17–84%, *p*-value <0.01) for one dose and 70% (95% CI: 28–88%, *p*-value 0.04) for two doses of MCV. There was no difference in VE by sex, availability of vaccination cards, and migrant status in both age groups (Table 3).

**Table 3.** Effectiveness of MCV in the under-5 and 5–15-year age groups by dose, stratified by sex, availability of vaccination card, and place of permanent residence, Eastern Mumbai slum, India

Person characteristics		Cases (%)	Controls (%)	Odds ratio (95% CI)	VE (%) (95% CI)	*p*-value
Under–5 age group						
One dose						
Overall		87 (58)	54 (32.7)	0.60 (0.38–0.95)	40 (5–62)	
Sex	Male	49 (56.3)	21 (38.9)	0.58 (0.30–1.12)	42 (0–70)	0.95
	Female	38 (43.7)	33 (61.1)	0.6 (0.30–1.17)	40 (0–70)	
Vaccination card availability	Available	22 (25.3)	22 (43.1)	0.5 (0.22–1.17)	50 (0–78)	0.24
	Not available	65 (74.7)	31 (60.8)	0.94 (0.5–1.72)	6 (0–50)	
Permanent residence	Mumbai	13 (14.9)	11 (20.4)	0.2 (0.05–0.75)	80 (25–95)	0.08
	Outside	74 (85.1)	43 (79.6)	0.7 (0.43–1.16)	30 (0–57)	
Two doses (among eligible)						
Overall		63 (42)	111 (67.3)	0.46 (0.27–0.77)	64 (23–73)	
Sex	Male	32 (51.6)	69 (68.3)	0.36 (0.17–0.77)	64(23–83)	0.35
	Female	30 (48.4)	42 (37.2)	0.60 (0.29–1.2)	40 (0–71)	
Vaccination card availability	Available	25 (39.7)	71 (64.0)	0.36 (0.14–0.91)	64 (9–86)	0.47
	Not available	38 (60.3)	40 (36.0)	0.74 (0.37–1.5)	26 (0–63)	
Permanent residence	Mumbai	4 (5.5)	16 (14.4)	0.17 (0.04–0.77)	83 (23–96)	0.16
	Outside	69 (94.5)	95 (85.6)	0.53 (0.30–0.93)	58 (26–77)	
5–15–year age group^a^						
One dose						
Overall		15 (31.2)	15 (15.2)	0.36 (0.16–0.83)	64 (17–84)	
Sex	Male	10 (66.7)	11 (73.3)	0.30 (0.10–0.85)	70 (15–90)	0.71
	Female	5 (33.3)	4 (26.7)	0.43 (0.10–1.80)	57 (0–99)	
Permanent residence	Mumbai	2 (13.3)	3 (20)	0.44 (0.04–5.10)	66 (0–96)	0.99
	Outside	13 (86.7)	12 (80)	0.43 (0.17–1.07)	57 (0–87)	
Two doses (among eligible)						
Overall		33 (68.8)	84 (84.8)	0.30 (0.12–0.72)	70 (28–88)	
Sex	Male	16 (48.5)	52 (61.9)	0.21 (0.07–0.67)	79 (33–93)	0.61
	Female	17 (51.5)	32 (38.1)	0.35 (0.07–1.70)	75 (0–93)	
Permanent residence	Mumbai	4 (12.1)	34 (40.5)	0.30 (0.02–1.07)	70 (0–98)	0.86
	Outside	29 (87.9)	50 (59.5)	0.38 (0.14–1.00)	62 (0–86)	

a Vaccination card was unavailable for the above-5 age group; hence, the stratum was excluded from the analysis.

## Discussion

We report an outbreak of measles in a slum area of Eastern Mumbai where a high proportion of eligible children missed either one or two doses of MCV, resulting in a widespread outbreak with a propagated mode of spread. The VE was low, albeit higher for two doses among all age groups, with the child’s sickness being the main reason for not receiving vaccination.

Globally, the progress towards measles elimination substantially declined in 2020 due to suboptimal measles surveillance and missed vaccination [13]. According to the World Health Organization (WHO), nearly 40 million children missed MCV dose(s) in 2021 leaving them highly vulnerable to infection, leading to an estimated nine million cases and 128,000 deaths globally.^14^ India was next only to Nigeria among nations that contributed to the highest number of infants who missed their first dose of MCV in 2020 [14].

A large proportion of children missing their MCV dose(s) indicate the problem of failure to vaccinate, which could have resulted in the accumulation of susceptible children in the slum area. This is corroborated by higher vaccination rates among the controls. The occurrence of 38% of cases among children aged > = 3 years also suggests suboptimal coverage of routine immunization in the area. The restrictions imposed as a result of the COVID-19 pandemic may have led to an accumulation of susceptible population in the community at large. The restrictions ranged from social distancing norms, which deterred parents from bringing children for immunization to programme-related factors including suspension of mass vaccination campaigns and disruption of the supply chain of vaccines, in addition to the diversion of human resources towards pandemic control leading to delayed or missed vaccination, thereby creating a suitable milieu for the outbreak [15, 16]. Since overcrowding facilitates transmission, the population living in the slums was particularly vulnerable. Vaccine hesitancy, influenced by religious, social, cultural, and personal factors, may have also played a role [17, 18]. A recent study by Tamysetty et al. in Eastern Mumbai’s slums highlighted concerns about vaccine safety, dependence on family members for vaccination decisions, fear, and lack of faith in immunization in the context of COVID-19 vaccination [19], some of which may be applicable to hesitancy to measles vaccination as well. Childhood sickness as the most commonly reported reason for missed vaccinations could be a socially desirable response implicitly suggesting vaccine hesitancy in this population. In addition to the above-mentioned factors, a common social scenario comprising large family size, lower family income, and children of working mothers increases the chances of missed vaccination among children residing in slums [20–22].

Poor VE can result from failure to vaccinate [23]. A seropositivity of 80–85% is reported in children who receive two doses of the MCV long after the vaccination [24], and the interval between the doses does not seem to affect the sero-protection [25]. The VE estimated in our study is much lower than the expected based on global estimates of MCV1 VE of 77% for the Southeast Asian region based on a meta-analysis [26]. This could be due to lower age at administration of measles vaccine [27] and programme-related factors, including suboptimal vaccine storage, handling, and administration [28]. Potentially inaccurately documented dose due to recall bias could have led to low estimates of VE in our study. Further implementation research is needed to understand the determinants of low VE factors and potential strategies to improve them in the Indian context.

Vaccine failure can be primary or secondary depending on whether or not the individual ever developed an immune response to the vaccine and can be host-related (immunodeficiency, age-related, malnutrition) [29–31] or vaccine-related (incomplete coverage of strains, intramuscular route of administration rather than subcutaneous, manufacturing differences) [32]. Children with secondary vaccine failure might be those who initially received and responded to the MCV, and over time, their protective immunity diminished, rendering them susceptible to the disease once again [25]. However, this needs to be further investigated by humoral and cell-mediated immune responses to identify whether vaccine failure was primary or secondary [33, 34].

It was observed during the outbreak that measles cases occurred among vaccinated children, which suggests the possibility of vaccine failure in addition to failure to vaccinate as a possible cause of this outbreak. The VE was particularly low among the under-5 population who had received only one dose of MCV, suggesting possible primary vaccine failure among those who had not received the second dose of MCV. Though we do not have primary data from the outbreak area, undernutrition as high as 25–30% is known to exist among the urban slum population in Mumbai [35], which might be one of the factors responsible for poor VE, resulting from frequent infections and consequent missed vaccination as well as vaccine failure. The prevalence of known risk factors for secondary vaccine failure, such as malnutrition, and the presence of other comorbidities need to be assessed in this area. These factors for secondary vaccine failure are relevant for children who had received both doses of MCV.

Our study has certain limitations. Firstly, we could not achieve the desired sample size in the 5–15-year age group, leading to a lower power in estimating the VE in this age group. Secondly, the vaccination status, wherever vaccination cards were not available, was based on recall by nearly half of the study participants in the under-5 and all of the 5–15-year age groups, and more so among the cases than the controls. This could have led to differential misclassification in vaccination status, resulting in the VE estimate biased in either direction. Thirdly, a measles infection prior to 90 days and conferred immunity thereof could have influenced the VE estimates. Finally, the study was conducted in a slum area in Mumbai; hence, the VE estimates cannot be generalized to the entire city.

In order to achieve elimination status, measles vaccine coverage of 95% with two doses is recommended by the WHO [36]. To regain momentum towards the 2023 elimination goal, catch-up vaccination efforts through government strategies are necessary to improve vaccine coverage wherever deficient. In addition to improving coverage, it may also be prudent to explore if a policy decision by the Indian government for additional vaccination at a later age will aid in improving the overall immune status of the community.

In order to prevent future outbreaks, improving vaccination coverage through targeted community awareness and outreach programmes and focusing on tracing and vaccinating children who missed doses due to illness need to be strengthened. Increased community engagement, involving religious leaders and influencers, can help build trust. In-depth research for understanding reasons for non-vaccination, a multi-stakeholder analysis of gaps in the implementation of the immunization programme, and the evaluation of the immunization programme in Eastern Mumbai, particularly in slum areas, can help inform future strategies to reduce missed opportunities for vaccination.

Outbreak response immunization and additional routine vaccination camps to complete the immunization of missed and dropout children were carried out by Mumbai civic authorities. It is worth noting that there was a delay in initiating the outbreak response immunization in the area, which could have possibly prevented such a large outbreak. All practitioners in the area, private hospitals, and nursing homes were informed to notify early of all cases of fever with rash irrespective of age. They were also instructed to isolate and adequately treat the cases and administer vitamin A to all cases of fever with rash to prevent complications. All school headmasters were instructed to report any case of suspected measles and to restrain such students from attending school to limit the spread of measles. Meetings with religious leaders were conducted to improve vaccine hesitancy resulting from religious grounds. The last case of measles was reported on 17 January 2023.

## Data Availability

Data supporting this study can be obtained upon reasonable request from the author.
